# Supplementary material for: AadT, a new weapon in Acinetobacter’s fight against antibiotics
Source: Microbiology (Reading). 2023 May 30;169(5):001341. doi: 10.1099/mic.0.001341 (PMC10268838; doi:10.1099/mic.0.001341)
Supplement: Supplementary material 1 [file mic-169-1341-s001.pdf]

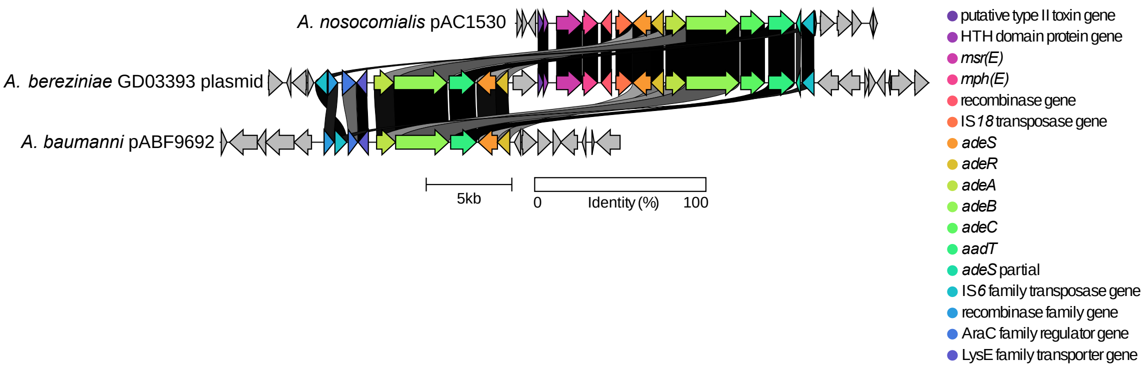

Figure S1. Local genetic organisation of the *aadT* loci in *A. bereziniae* GD03393 plasmid. One gene cluster shows high sequence similarity to the *aadT* locus in *A. nosocomialis* AC1530 pAC1530 and an adjacent cluster shows high similarity to the *aadT* locus in the *Acinetobacter baumannii* pABF9692. This figure was made using clinker v0.0.25 (1).

Reference

1. Gilchrist CLM, Chooi YH. Clinker & clustermap.js: Automatic generation of gene cluster comparison figures. Bioinformatics. 2021.
