# Supplementary material for: AadT, a new weapon in Acinetobacter’s fight against antibiotics
Source: Microbiology (Reading). 2023 May 30;169(5):001341. doi: 10.1099/mic.0.001341 (PMC10268838; doi:10.1099/mic.0.001341)
Supplement: Supplementary material 2 [file mic-169-1341-s002.pdf]

	AadT pAL_065_2	AadT pABF9692	AadT *Can.* A. avistercoris MAG	AadT pAC1530	AadT *Pelagibacterium* MAG	AadT *Proteobacterium* MAG	*AmvA*	*SmvA*	*PqrB*	*CmgA*	*QacA*	*QepA*	*LfrA*	*SgvT1*	*RifP*	*Ptr*
**AadT pAL_065-2**		98	88.1	97	72.6	66.6	42.7	43.5	36.2	33.8	34.2	35.5	32.9	30.9	31.2	30.9
**AadT pABF9692**	99.2		89.1	99	72.4	67.2	42.9	43.5	36.4	33.5	34.4	36.2	32.8	30.6	31.6	31
**AadT *Can.* A. avistercoris MAG**	93.5	94.2		88.9	70.1	66.6	44.9	43.7	35.8	34.4	33	35.7	33.5	32.5	32.2	30.9
**AadT pAC1530**	98.6	99.4	94.2		72.4	67	42.7	43.5	35.8	33.6	33.8	36	32.2	30.4	31.2	31
**AadT *Pelagibacterium* MAG**	83.9	83.5	83.3	83.7		71.7	43	45.3	38	32.5	32.1	38.3	33.9	32	34.7	34.6
**AadT Proteobacteria MAG**	82.6	82.4	81.2	82.6	84.4		41.5	43.1	37.2	34.7	31.8	36.7	35.7	32	32.2	32
**AmvA**	66.5	66.1	67.5	66.3	65.3	62.6		55.2	33	32	28.7	30.7	33.6	27.9	31.2	31.6
**SmvA**	67.1	67.5	67.7	67.7	67.1	63.4	76.4		37.4	34.2	32.3	35.5	32.4	31.7	34.9	31.5
**PqrB**	57.1	57.1	54.9	56.9	56.5	57.1	59.4	59.2		39.7	36	45.8	47.8	40	40.4	38.1
**CmgA**	58.5	58.3	58.3	58.1	56.2	57.5	59.3	56.6	59.8		33	35.8	33.3	32.5	32.5	34.7
**QacA**	57.2	57.2	56.8	56.8	54.5	56	53.5	54.1	60.3	55.3		33.1	33.5	29.4	30.3	31.5
**QepA**	55.6	55.8	57.3	55.8	55.2	55.4	54.4	56.4	64.8	55.6	59.9		42.4	31.3	35.7	35.2
**LfrA**	54.4	54.2	53.2	53.8	53.4	55.7	58.5	54.8	69.2	54.6	57.8	63.4		34	33	31.4
**SgvT1**	52.6	52.5	55.3	53.4	53.2	52.6	52.1	51.3	59.4	53.8	52.8	53.6	53.2		50.3	48.2
**RifP**	52.9	53.1	53.3	52.9	54.2	53.3	54.4	53.6	59	51.5	55	57.1	53.8	68.5		62.5
**Ptr**	53.6	54.4	56.7	54.2	57.2	55.9	55.7	53.7	56.3	56.3	54.3	56.4	55.4	66.8	77.8	

Figure S2. Pairwise amino acid sequence similarity (bottom left) and identity (top right) scores for members of the DHA2 family of efflux proteins. These values were determined using MatGat version 2.0 (1) with a BOLSUM 50 scoring matrix.

Reference

1. Campanella JJ, Bitincka L, Smalley J. MatGAT: an application that generates similarity/identity matrices using protein or DNA sequences. BMC Bioinformatics. 2003;4:29.
